# Salt loading as a promising approach to study the dopaminergic phenotype of neurons of the supraoptic nucleus in mice

**DOI:** 10.1371/journal.pone.0340281

**Published:** 2026-02-06

**Authors:** Alyona E. Bannikova, Tatiana S. Pronina, Dmitry V. Troshev, Vsevolod V. Bogdanov, Anna A. Kolacheva, Ekaterina N. Pavlova, Victor E. Blokhin, Varvara I. Kalashnikova, Michael V. Ugrumov

**Affiliations:** Laboratory of Neural and Neuroendocrine Regulations, Koltzov Institute of Developmental Biology of the Russian Academy of Sciences, Moscow, Russia; University of Nebraska Medical Center College of Medicine, UNITED STATES OF AMERICA

## Abstract

Until the beginning of this century, neurons of the supraoptic nucleus (SON) were repeatedly shown to express tyrosine hydroxylase (TH) in salt loaded rats. However, its role remains unsolved due to methodological problems. Given that these issues can now be solved using transgenic mice and more advanced methods, the aim of this study was to reproduce the salt loading models used in rats, in C57BL/6 mice and transgenic mice expressing the green fluorescent protein gene under the TH promoter. Our study also attempted to identify a model that would most significantly increase TH synthesis in vasopressinergic neurons. This was assessed with immunocytochemistry by measuring the number of TH-immunoreactive neurons in the SON and the intraneuronal content of TH-immunoreactive material in individual neurons. In the first model, when using 3% NaCl as drinking water, the highest number of TH-immnopositive neurons was detected on the 3rd day, while the intraneuronal TH content did not change. In the second model, 10 hours after the intraperitoneal administration of 8.5% NaCl (experiment) or 0.9% NaCl (control), the number of TH-immunopositive neurons was significantly higher than in the first model. Moreover, the intraneuronal content of TH increased. Additional PCR analysis showed in the second model an increase in the expression of the TH gene and genes of some transcription factors (Sp1, Atf4, c-Fos, c-Jun) that initiate the TH gene expression in SON. Thus, we developed and characterized a salt loading model in mice with the highest level of TH synthesis, which will be used in the future to assess the functional significance of this protein.

## Introduction

Studies over the past 35 years have shown that in the hypothalamus, in addition to dopaminergic (DAergic) neurons expressing both DA-synthesizing enzymes and the dopamine transporter (DAT), there are numerous neurons partially expressing the DAergic phenotype [1]. Some neurons contain only the first enzyme of DA synthesis, tyrosine hydroxylase (TH), which converts L-tyrosine to L-dioxyphenylalanine (L-DOPA). Other neurons contain only the second enzyme of DA synthesis, aromatic L-amino acid decarboxylase, which converts L-DOPA to DA. L-DOPA is taken up into these neurons from the intercellular space by the transporter of large neutral amino acids [2]. While in the initial studies, it was believed that neurons partially expressing the DAergic phenotype are located only in the hypothalamus and that their number is rather low, it was eventually shown that such neurons are widespread throughout the brain and also located in the spinal cord [3,4]. It seems that the total number of neurons partially expressing the DAergic phenotype significantly exceeds the number of DAergic neurons.

From the physiological point of view, hypothalamic neurons expressing only TH are of particular interest. Indeed, monoenzymatic TH-expressing neurons of the arcuate nucleus and of the periventricular nucleus secrete L-DOPA, which is thought to play the role of a neurotransmitter, entering the neuropil, and the role of a neurohormone, entering the cerebrospinal fluid and the general circulation [5,6]. Of particular interest is the fact that the expression of DA-synthesizing enzymes in non-DAergic neurons of the hypothalamus increases or is triggered due to an increase in their functional activity, which is generally considered a compensatory reaction [7,8]. Indeed, degeneration of DAergic neurons in the arcuate nucleus and the resulting DA deficiency are accompanied by an increase in the expression of DA-synthesizing enzymes in this nucleus [7,9].

In the late 1980s, it was first shown in rats that TH is synthesized not only in neurons of the arcuate nucleus and periventricular nucleus of the hypothalamus, but also in much larger in size vasopressin (VP)ergic and oxytocin (OT)ergic neurons localized in the so-called magnocellular supraoptic (SON) and paraventricular nuclei [10]. Unlike neurons of the periventricular and arcuate nuclei, numerous neurons of the magnocellular nuclei of the hypothalamus synthesize TH, but only when they are functionally stimulated, primarily during salt loading, or denervation [10]. It is important to note that VPergic and OTergic neurons synthesizing TH have also been found in humans in norm and pathology (hypoxia, etc.) [11,12].

In the 1990s and early 2000s, numerous attempts were made to determine in salt loading rats: (i) to what extent VPergic and OTergic neurons have a DAergic phenotype, (ii) what is the functional role of TH in VPergic and OTergic neurons, and (iii) what is the role of intercellular signals in the regulation of TH gene expression and TH synthesis in these neurons [10,13,14]. However, these attempts were unsuccessful [15], and therefore, this research was suspended in the first decade of this century. Luckily, new sophisticated methodological approaches have been developed over the past 20 years that can help to solve the above problems. In particular, the use of transgenic mice opened up a fundamentally new opportunity to study the genetic, molecular and physiological mechanisms of brain function in general and in magnocellular neurons of the hypothalamus in particular. Indeed, in recent years, many studies of brain functions have been carried out not in rats, but in transgenic mice [16]. Based on this idea, it is advisable to move from the use of salt loading models with enhanced TH synthesis in magnocellular neurons in rats to the use of similar models in mice. The efficiency of such studies can also be improved due to the enhancement of physiological and molecular biological methods in recent decades [1].

Based on the above, the aim of this study was to reproduce in mice the salt loading models previously used in rats and to select a model with the highest number of TH-synthesizing neurons in the SON and the highest level of TH synthesis in individual neurons. This is important for further evaluation of the presumptive DAergic phenotype of magnocellular neurons, since the number of neurons in the magnocellular nuclei of the hypothalamus in mice is smaller than in rats [17,18]. When selecting the optimal model of salt loading in mice with increased TH expression in SON neurons, we primarily proceeded from the models that have been developed in rats. These were most often carried out by replacing drinking tap water with a NaCl solution with a concentration no more than 2% for 5–21 days [14,17]. Less frequently, an acute salt loading model was performed in rats by a single systemic injection of hypertonic NaCl solution (6% or 8.5%). In this case, the material was obtained over a period from 15 minutes to 24 hours after the injection [19,20]. A salt loading model using intraperitoneal administration of hypertonic NaCl solution (8.5%) has also been used previously in mice, but TH expression in SON neurons has not been assessed in these studies [18,21].

## Materials and methods

### Animals and experimental procedures

Male C57BL/6 mice (n = 104) and B6.B6D2-Tg(Th-EGFP)21–31Kobа transgenic mice (n = 8) (RIKEN BRC, Tsukuba-shi, Ibaraki, Japan) at the age 8–12 weeks weighing 20–25 g were used in this study. C57BL/6 mice were obtained from the Stolbovaya breeding center (SCBMT RAMS, Stolbovaya, Moscow region, Russia). B6.B6D2-Tg(Th-EGFP)21–31Kobа transgenic mice, in which the expression of the green fluorescent protein (GFP) gene is carried out under the control of the TH gene promoter [22], were bred by crossing the B6.B6D2-Tg(Th-EGFP)21–31Kobа mice with inbred mice of the C57BL/6 strain. The offspring was genotyped by amplification of tail tissue genomic DNA according to the RIKEN BRC protocol (Sheet ID PS_05058).

The animals were kept at a temperature of 22 ± 1 °C, with a 12-hour day/night cycle and free access to food and water. Experimental procedures were conducted in accordance with the National Institutes of Health Guide for the Care and Use of Laboratory Animals (8th edition, 2011) and were approved by the Animal Care and Use Committee of the Koltzov Institute of Developmental Biology of the Russian Academy of Sciences (protocol No. 85 from 09/05/2024).

**Fig 1:**
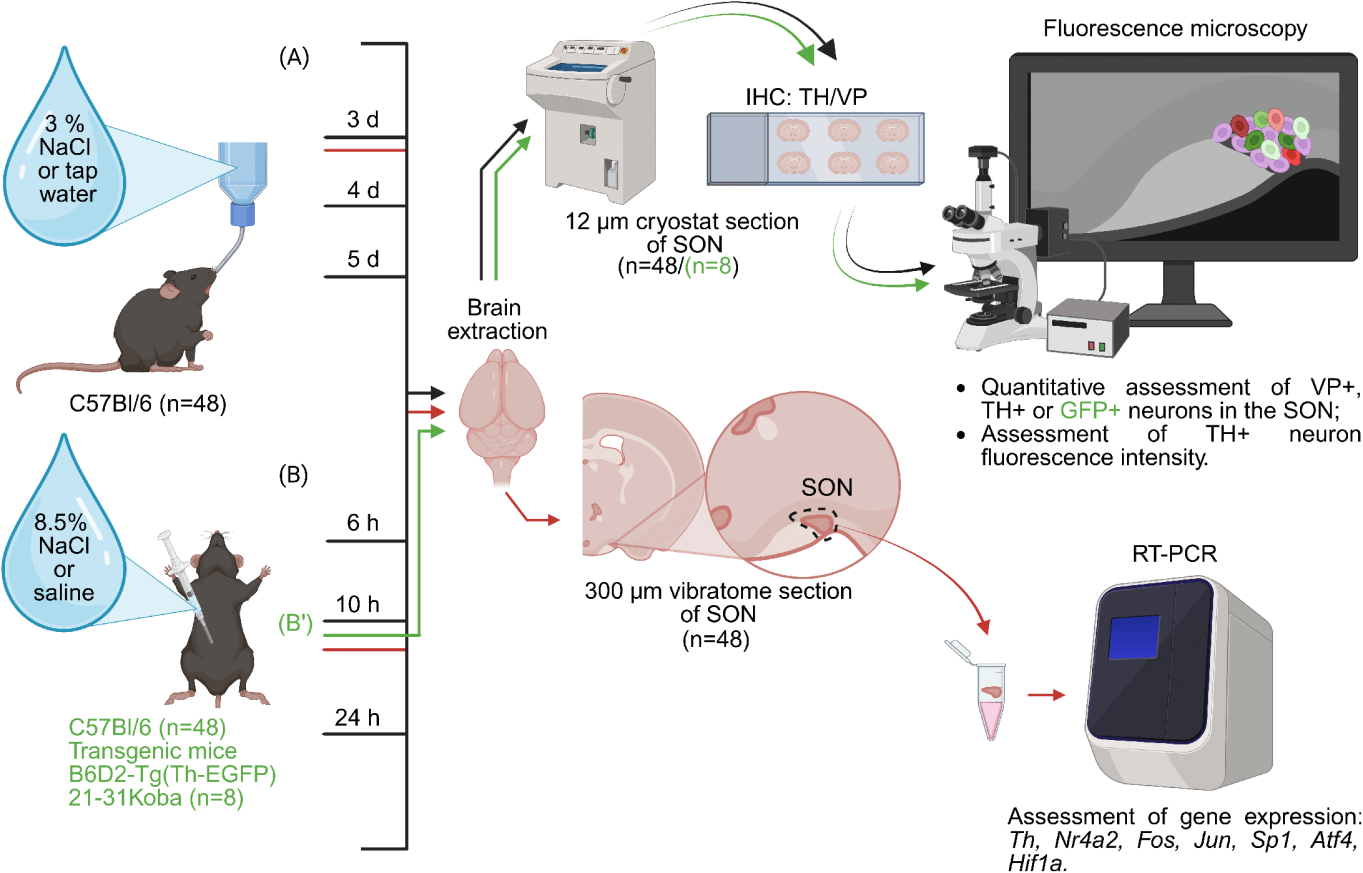
Schematic representation of salt loading experiments in C57BL/6 mice and B6.B6D2-Tg(Th-EGFP)21-31Koba transgenic mice: (A) Drinking 3% NaCl by C57BL/6 mice for 3, 4, or 5 days followed by assessment of changes in the number of vasopressin (VP)- and tyrosine hydroxylase(TH)-immunopositive neurons in the supraoptic nucleus (SON) (black arrows) (n = 24) at all indicated time points, as well as changes in TH gene expression (red arrows) (n = 24) and TH immunofluorescence intensity (n = 8) 3 days after the onset of the experiment compared to the control (drinking tap water). (B) A single intraperitoneal injection of 8.5% NaCl in the experiment and 0.9% NaCl in the control, followed by an assessment of the change in the number of VP- and TH-immunopositive neurons in the SON 6, 10, and 24 hours after injection of 8.5% NaCl compared to the control (saline) at the same time points (black arrows) (n = 24), as well as a change in TH gene expression (red arrows) (n = 24) and TH immunofluorescence intensity (n = 8) 10 hours after 8.5% NaCl injection compared to the control (saline). (B’) Evaluation of the number of VP-, TH-, GFP-, and TH/GFP-positive neurons in the SON of B6.B6D2-Tg(Th-EGFP)21-31Koba mice (green arrows) (n = 8) 10 h after intraperitoneal injection of 8.5% NaCl compared to the controls – intraperitoneal injection of 0.9% NaCl. d – day, h – hour, TH+ tyrosine hydroxylase-immunopositive, GFP + – green fluorescent protein-immunopositive, VP+ vasopressin – immunopositive neurons.

Animals of the experimental group were administered hypertonic NaCl solution in two ways (Fig 1). In the first experimental group (n = 24), C57BL/6 mice drank 3% NaCl solution dissolved in tap water for 3, 4, and 5 days (Fig 1A), whereas control mice drank tap water for the same period (n = 24). In the second experimental group, C57BL/6 mice (n = 24) were given a single intraperitoneal injection 30 ml/kg of 8.5% NaCl (Sigma, USA) [28]. Mice in the control group (n = 24) were given a single intraperitoneal injection 30 ml/kg of 0.9% NaCl. After the injection, mice in the experimental and control groups were kept for 6, 10, or 24 hours at 22 ± 1 °C, with a 12 h day/night cycle and free access to food, but with no access to water (Fig 1B). B6.B6D2-Tg(Th-EGFP)21–31Kobа mice (n = 4 per group) were given a single intraperitoneal injection of 8.5% NaCl in the experimental group and 0.9% NaCl in the control, with subsequent material collection 10 hours after the injection (Fig 1B’).

Mice of the experimental group and control group were decapitated under anesthesia with 2.4% isoflurane (Laboratorios Karizoo, Spain) using an anesthesia device SomnoSuite (Kent Scientific, USA), and the brains were removed from the skulls. Some brains were used for PCR (see “Methods. RNA extraction from the supraoptic nucleus and quantitative polymerase chain reaction”). For this purpose, they were placed into cold Krebs-Ringer solution (120 mM NaCl, 4.8 mM KCl, 2 mM CaCl_2_, 1.3 mM MgSO_4_, 25 mM NaHCO_3_, 10 mM D-glucose, 20 mM HEPES, and 0.1 mM ascorbic acid (all from Sigma-Aldrich, USA), pH = 7.2). Frontal brain sections with a thickness of 300 µm were prepared using a vibratome (Leica VT1200S, Leica Biosystems, Germany). The SON was cut out from both hemispheres of the brain from two vibratome sections with razor blades under the control of a binocular light microscope (Leica Camera AG, Germany). The resulting samples were frozen in liquid nitrogen and stored at −70 °C until RNA extraction (n = 12 per group).

Other brains were used for morphological studies. In this case, the material was fixed in 4% paraformaldehyde (Sigma, USA), prepared in a 0.1 M phosphate buffer (pH = 7.2–7.4) for 12 hours at 4 °C. The brains were then washed in 0.02 M phosphate-buffered saline (PBS) (pH = 7.2–7.4), incubated in 20% sucrose in 0.02 M PBS for 24 hours at 4 °C, and frozen in hexane cooled to −40 °C. The samples were stored at −70 °C until immunostaining for VP and TH I n SON sections (n = 4 per group).

## Methods

### Immunohistochemistry

For immunohistochemical study, SON frontal sections with a thickness of 12 µm were prepared from the frozen brains of experimental and control animals (n = 4 per group) over the distance from −0.46 mm to −1.06 mm in the rostro-caudal direction relative to Bregma in accordance with the mouse brain atlas [23] using a cryostat (Leica CM1950, Leica, Wetzlar, Germany). Every third SON section was mounted onto a glass slide.

Sections were immunostained for both TH and VP (double immunostaining, see S1 File). To do this, they were sequentially incubated in PBS containing: (i) 3% bovine serum albumin (Sigma, USA) and 0.3% Triton X-100 (Sigma, USA) for 1 hour at 20 °C; (ii) sheep polyclonal antibodies to TH (1:700) (Millipore, USA, AB 1542), rabbit monoclonal antibodies to VP (1:500) (Abcam, USA, AB 213708), 1% bovine serum albumin, and 0.1% Triton X-100 for 20 hours at 20 °C; (iii) Alexa-Fluor-555 donkey antibodies against rabbit gamma globulins (1:1000) (Invitrogen, USA, A32794) for 2 hours at 20 °C; and (iv) Alexa-Fluor-488 donkey antibodies against sheep gamma globulins (1:1000) (Invitrogen, USA, A11015) for 2 hours at 20 °C. After each incubation, except for the first, the sections were washed three times in PBS for a total of 45 minutes. After the last incubation, the sections were washed in PBS for an hour and mounted into a medium containing DAPI (Abcam, USA).

For double-immunostaining of TH and VP in SON neurons in B6.B6D2-Tg(Th-EGFP)21–31Koba mice (n = 4 per group), we used the method described above except for step “iv”, where Alexa-Fluor-633 donkey antibodies against sheep gamma globulins (1:500) (Invitrogen, USA, A21100) were used. To assess colocalization of GFP and immunostained GFP in SON neurons in B6.B6D2-Tg(Th-EGFP)21–31Koba mice, we used the protocol described above for transgenic mice, but with immunostaining using rabbit polyclonal antibodies to GFP (1:500) (Evrogen, Moscow, Russia) instead of VP.

### Microscopy and quantitative image analysis

The SON neurons immunostained for TH and VP were examined under a fluorescence microscope (Leica DMi8 M, Leica Camera AG, Wetzlar, Germany) at 20x objective magnification (The Core Centrum of the Koltzov Institute of Developmental Biology RAS).

The total number of neuron cell bodies (further – neurons) immunopositive for TH was counted on every third SON section in C57BL/6 and B6.B6D2-Tg(Th-EGFP)21–31Koba mice. In addition, the number of neurons labeled for GFP and the number of neurons immunostained simultaneously for TH and GFP were counted in transgenic mice. Immunostained neurons were counted on every third section from the complete SON section series. To avoid double counting of neurons, the Abercrombie formula was used [24]:


$$N=\sum_{i=\text{1}}^{i}\left(ni\cdot (Si+\text{1})\cdot \frac{h}{h+D}\right)$$

N, the total number of neurons;

ni, the number of neurons per selected section;

Si, the number of skipped sections;

h, section thickness, in μm;

D, neuron diameter, in μm.

### Semi-quantitative immunocytochemical analysis of tyrosine hydroxylase-immunopositive material in neurons of the supraoptic nucleus

The fluorescence intensity of the TH-immunoreactive material was assessed on the same photographs that were used to count the SON neurons [25]. The images were converted to 8-bit format using FiJi software (see, https://imagej.net/software/fiji/downloads). The “Mean gray value” parameter was used to estimate the fluorescence intensity of TH-immunoreactive material in the cytoplasm. This was done after manual contouring of neurons and their nuclei obtained after manually tracing the cells (avoiding the nucleus), as well as three randomly selected zones within the section without specific immunostaining, to measure the “background”. Mean gray values/µm^2^ were compared with each other within each “pair” of control and experimental sections of the SON for each experimental group. A “pair” was considered to be sections of the control and experimental groups, placed on the same glass and processed under the same conditions.

### RNA extraction from the supraoptic nucleus and quantitative polymerase chain reaction

RNA was extracted from SON samples using 1 ml of TRI-reagent (MRC Inc., Cincinnati, OH, USA). 100 μl of 1-bromo-3-chloropropane (Sigma-Aldrich, USA) was added to the TRI-reagent, the mixture was vortexed and incubated for 15 minutes at 20 °C. The phases were then separated by centrifugation for 15 minutes at 21,000 × g and 4 °C. The aqueous phase containing RNA was transferred to a new tube, 500 μl of isopropyl alcohol (Sigma-Aldrich, USA) and 0.5 μl of glycogen (Thermo Fisher Scientific, USA) were added. The solution was vortexed and incubated for 10 min at 20 °C, after which the RNA was precipitated by centrifugation for 10 min at 21,000 × g and 4 °C. The supernatant was removed, the precipitate was washed twice with 1 ml of 80% ethanol and centrifuged for 10 minutes at 21,000 × g and 4 °C. After the last centrifugation, the ethanol was removed and the RNA precipitate was dried for 15 minutes. The RNA concentration in all samples was measured using a NanoDrop 8000 (Thermo Fisher Scientific, USA). The remaining genomic DNA was removed using DNase I RNase-free (Thermo Fisher Scientific, USA) according to the manufacturer’s recommendations. Complementary DNA was synthesized from 300 ng of RNA using the MMLV RT synthesis kit (Evrogen, Moscow, Russia) according to the manufacturer’s recommendations. PCR was performed on a QuantStudio 12k Flex thermocycler (Applied Biosystems, Waltham, USA) using the qPCRmix-HS SYBR + LowROX reaction mixture (Evrogen, Moscow, Russia) and oligonucleotide primers (Evrogen, Moscow, Russia) presented in Table 1.

**Table 1:** Oligonucleotide primers used for quantitative polymerase chain reaction.

Gene	Protein	Forward primer	Reverse primer
*Cyc1*	Cytochrome C1	GCGGCCAGGGAAGTTGT	GCCAGTGAGCAGGGAAAATAC
*Th*	Tyrosine hydroxylase	TCAGAGGAGCCCGAGGTC	GGGCGCTGGATACGAGAG
*Avp*	Arginine vasopressin	CCCAAGAGGCGGCAAGAG	CAGGGCGAGGGCAGGTAG
*Nr4a2*	Transcription factor Nurr1	*CCGAAGAGCCCACAGGAT*	*CCATAGAGCCGGTCAGGAG*
*Fos*	c-Fos	*AGAGCGCCCCATCCTTAC*	*GCTCTACTTTGCCCCTTCTG*
*Jun*	c-Jun	*CGCCCCTGTCCCCTATC*	*TAAGCTGTGCCACCTGTTCC*
*Sp1*	Transcription factor Sp1	*GGCCTTGCTAATAATGTGCTCT*	*CATGTTGCTGGTGGTAGTAGTTGT*
*Atf4*	Activating transcription factor 4	*CTTATGACCCACCTGGAGTTAGT*	*CTAGTGGCTGCTGTCTTGTTTT*
*Hif1a*	Hypoxia-inducible factor 1-alpha	*ACATGATGGCTCCCTTTTTC*	*CTCCGTTCCATTCTGTTCACT*

Gene expression was assessed using the 2^–ΔΔCt^ method (see S2 File). *Cyc1* was used as a housekeeping gene. *Avp* expression level was used to determine the accuracy of SON dissection from the brain sections as recommended [26]. Samples with the lowest *Avp* expression levels were excluded from the analysis. The expression of transcription factor genes was determined in mice 10 hours after intraperitoneal administration of 8.5% NaCl in the experiment and 0.9% NaCl in the control, as well as 3 days after drinking 3% NaCl in the experiment and tap water in the control (n = 12 per group).

### Statistics

Statistical analysis was performed using the GraphPad Prism 9 software (GraphPad Software, San Diego, CA, USA). The type of distribution was assessed using the Shapiro-Wilk test. For pairwise comparisons, the paired t-test was used. For parametric multiple comparisons, one-way ANOVA and post-hoc Tukey test were used. For nonparametric pairwise multiple comparisons, Dunn’s test was used. The results are presented as mean ± SEM or as mean with interquartile range. Differences were considered significant at p ≤ 0.05.

## Results

### The number of vasopressin and tyrosine hydroxylase-immunopositive neurons in the supraoptic nucleus of C57BL/6 mice after salt loading

**Fig 2:**
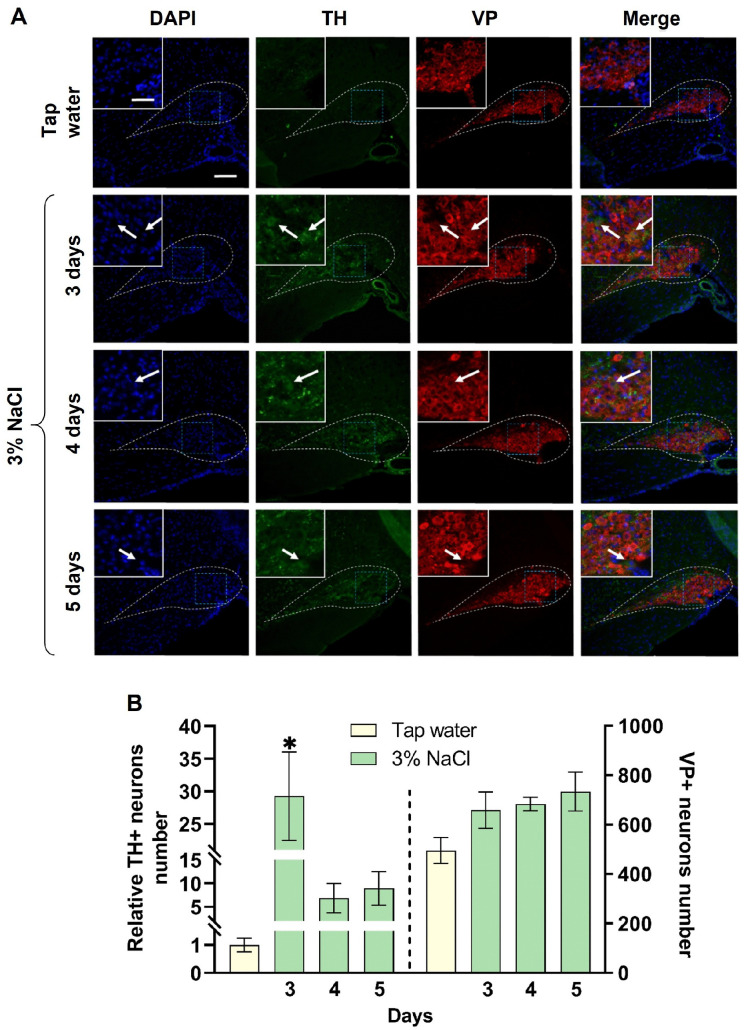
Vasopressin(VP)- and tyrosine hydroxylase(TH)-immunopositive neurons in the supraoptic nucleus (SON) of C57BL/6 mice after drinking of 3% NaCl for 3, 4, and 5 days (experiment) and tap water (control). (A) TH-immunopositive (green) and VP-immunopositive (red) neurons with nuclei stained with 4′,6-diamidino-2-phenylindole (DAPI, blue). White dotted line – SON border, blue dotted line – fragment of the same section at higher magnification (upper left corner), white arrow – TH-immunopositive neuron. Scale bars: main image – 100 µm; magnified area – 20 µm. (B) The number of TH-immunopositive (TH+) neurons in relation to the control group and the absolute number of vasopressin-immunopositive (VP+) neurons in the SON 3, 4, and 5 days of drinking 3% NaCl in the experiment and tap water in the control (n = 4 per group). The groups were compared for normality by using the Shapiro-Wilk test. The groups were compared using Dunn’s test for multiple comparisons, where * corresponds to statistically significant differences with control group at p ≤ 0.05. The data are presented as median with interquartile range.

In the mice of the first experimental group, 3 days after drinking 3% NaCl, the SON contained 168 ± 39 TH-immunopositive neurons, whereas in the control animals that received tap water, the SON contained 6 ± 1 TH-immunopositive neurons, i.e., 29.2 times less (p = 0.02). After 4 and 5 days of drinking 3% NaCl, the number of TH-immunopositive neurons in the SON did not change compared to the control (Fig 2).

In mice of the control animals that received tap water, the SON contained 495 ± 52 VP-immunopositive neurons. In the experimental groups, after 3, 4, and 5 days of drinking 3% NaCl, the number of VP-immunopositive neurons was the same as in the control (Fig 2).

**Fig 3:**
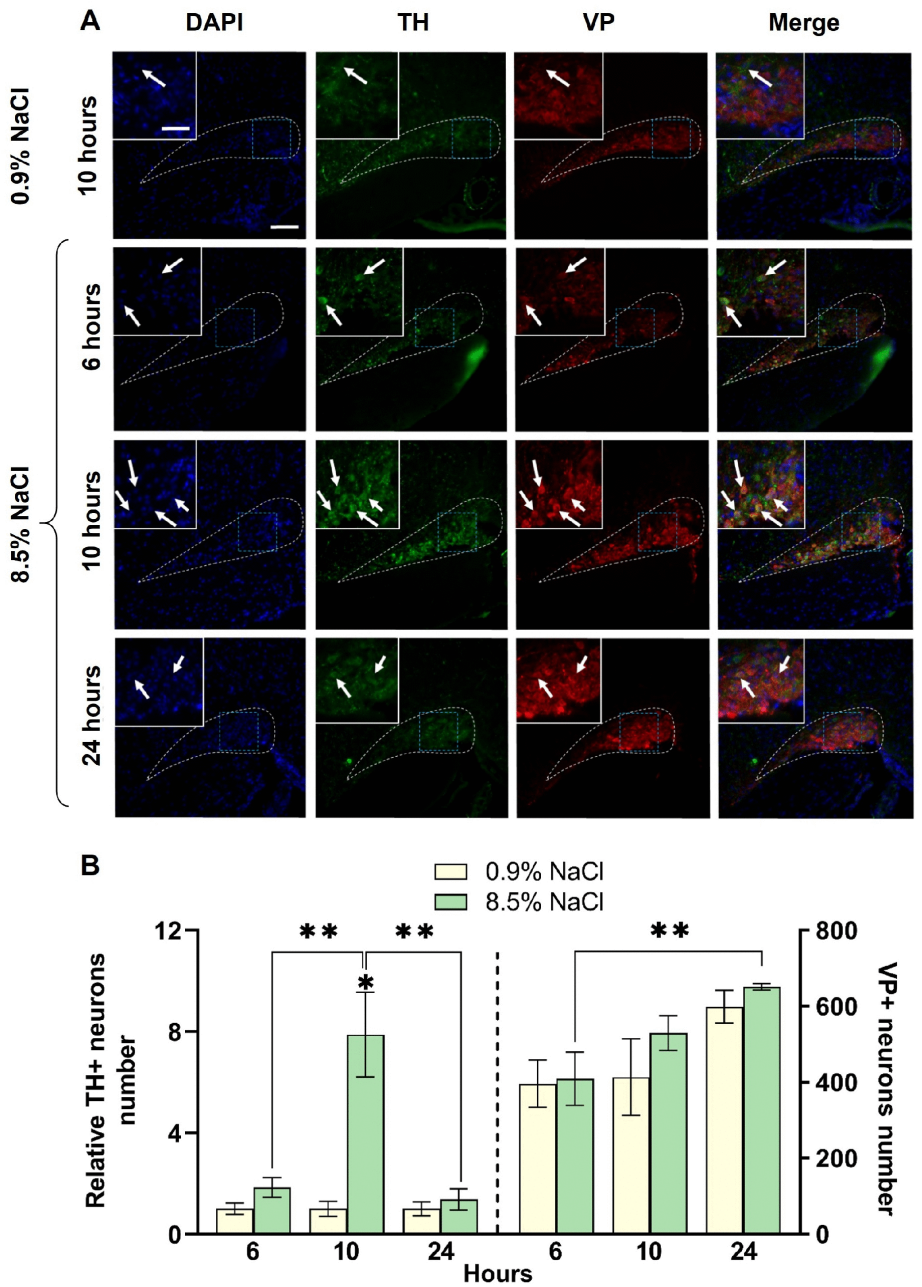
Vasopressin(VP)- and tyrosine hydroxylase(TH)-immunopositive neurons in the supraoptic nucleus (SON) of C57BL/6 mice 6, 10, and 24 hours (h) after a single intraperitoneal injection of 8.5% NaCl. (A) TH-immunopositive (green) and vasopressin(VP)-immunopositive (red) neurons with nuclei stained with 4′,6-diamidino-2-phenylindole (DAPI, blue) in the SON of C57BL/6 mice. White dotted line – SON border, blue dotted line – fragment of the same section at higher magnification (upper left corner), white arrow – TH-immunopositive neuron. Scale bars: main image – 100 µm; magnified area – 20 µm. (B) The number of TH-immunopositive (TH+) neurons in relation to the control group and the absolute number of vasopressin-immunopositive (VP+) in the SON 6, 10, and 24 hours after a single intraperitoneal injection of 8.5% NaCl in the experiment and 0.9% NaCl in the control (n = 4 per group). The groups were compared for normality by using the Shapiro-Wilk test. Statistical analysis of multiple comparisons was performed using the one-way ANOVA and post-hoc Tukey’s test, where * corresponds to statistically significant differences with control group at p ≤ 0.05 and ** corresponds to statistically significant differences between selected parameters. The data are presented as mean ± SEM.

In mice of the second experimental group, 6 hours after a single intraperitoneal injection of 8.5% NaCl, the number of TH-immunopositive neurons in the SON did not change compared to the control (0.9% NaCl) (127 ± 27 vs. 69 ± 15, p = 0.9639) (Fig 3). Ten hours after the intraperitoneal injection of 8.5% NaCl, the SON contained 290 ± 62 TH-immunopositive neurons, while in the control (0.9% NaCl) it contained 54 ± 8 neurons. It means that in the experiment, there were 7.8 times as many TH-immunopositive neurons (p < 0.0001) compared to the control. Twenty four hours after the intraperitoneal injection of 8.5% NaCl, no significant difference (p = 0.9991) was observed in the number of TH-immunopositive neurons in the SON in the experiment, compared with the control (Fig 3).

In mice of control groups that received intraperitoneal injections of saline, the number of VP neurons did not change. In the experimental groups of mice that received intraperitoneal injections of 8.5% NaCl, the number of VP neurons increased from 409 ± 70 6 hours after injection to 651 ± 8.6 24 hours later. However, no differences were observed in the number of VP-immunopositive neurons between the experimental and control groups at any time studied (Fig 3).

### Tyrosine hydroxylase content in the neurons of the supraoptic nucleus of C57BL/6 mice after salt loading

**Fig 4:**
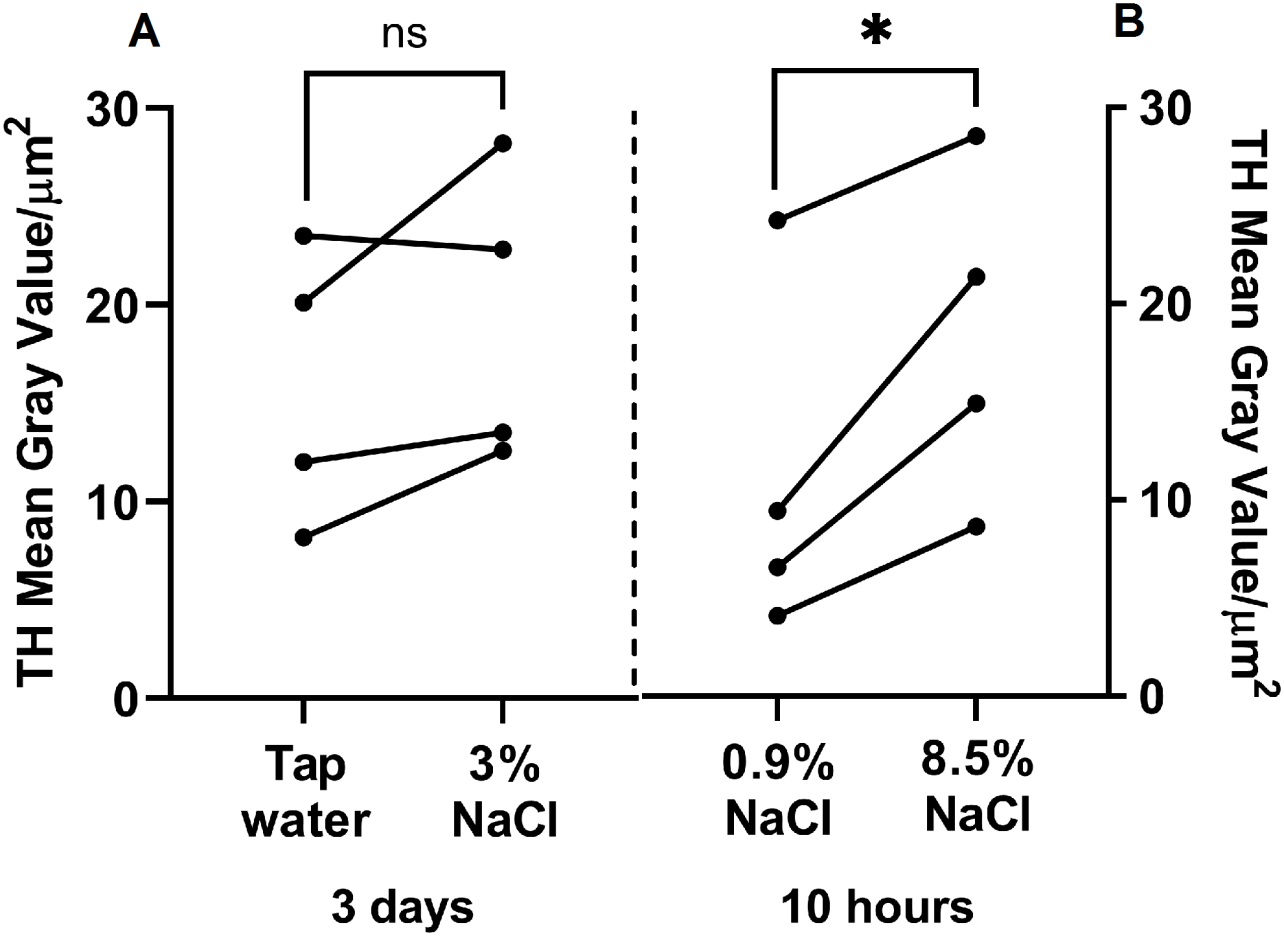
Fluorescence intensity of tyrosine hydroxylase (TH)-immunopositive neurons (mean gray value) in the supraoptic nucleus (SON) of C57BL/6 mice after osmotic stimulation. (A) Animals drank 3% NaCl for 3 days in the experiment and tap water for the same time in the control; (B) animals 10 hours after a single intraperitoneal injection of 8.5% NaCl in the experiment and 0.9% NaCl in the control (n = 4 per group). d – day, h – hour. Groups were compared for normality using the Shapiro-Wilk test. Statistical analysis was performed using the paired t-test, where * corresponds to statistically significant differences at p ≤ 0.05. Data are presented as mean ± SEM.

In the first experimental group, in which the tap water was replaced with 3% NaCl for 3 days, the fluorescence intensity of TH-immunopositive neurons did not differ (p = 0.1790) from that of mice in the control group that received tap water (Fig 4A).

In the second experimental animal group, 10 hours after a single intraperitoneal injection of 8.5% NaCl, the fluorescence intensity of TH-immunopositive neurons was twice as high (p = 0.0276) as in the animals of the control group received 0.9% NaCl (Fig 4B).

### Tyrosine hydroxylase gene expression in the neurons of the supraoptic nucleus in C57BL/6 mice after salt loading

**Fig 5:**
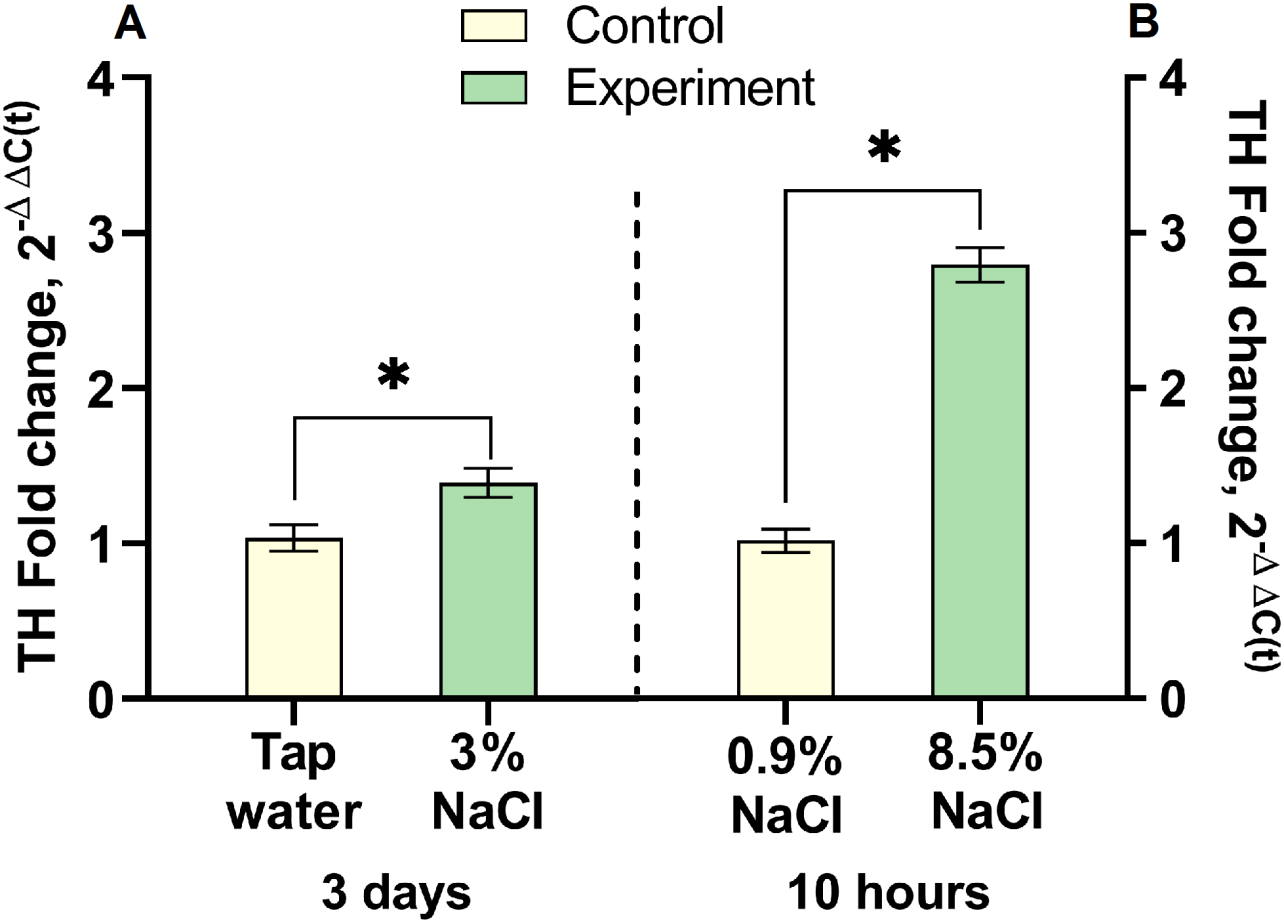
*Th* expression in the supraoptic nucleus (SON) of mice under salt load. (A) Difference in *Th* expression in SON neurons after drinking 3% NaCl for 3 days compared to control animals drinking tap water for the same period. (B) Difference in *Th* expression in SON 10 hours after a single intraperitoneal injection of 8.5% NaCl in the experiment and 0.9% NaCl in the control (n = 12 per group). The relative level of *Th* expression was estimated by the 2^-ΔΔC(t)^ method. The level of gene expression in the control was taken as 1. The groups were compared for normality by using the Shapiro-Wilk test. Statistical analysis was performed using the paired t-test, where * corresponds to statistically significant differences at p ≤ 0.05, compared with the control. The data are presented as mean ± SEM.

In the first experimental group, in which mice drank 3% NaCl for 3 days, *Th* expression increased 1.3 folds (p = 0.0114) compared to the control – mice drank tap water for the same time (Fig 5A). In the second experimental group – 10 hours after a single intraperitoneal injection of 8.5% NaCl to mice, *Th* expression was 2.74 times higher (p < 0.0001) than in the control (0.9% NaCl) (Fig 5B).

### Сolocalization of endogenous green fluorescent protein and green fluorescent protein-immunopositive material in neurons of the supraoptic nucleus of transgenic mice after salt loading

**Fig 6:**
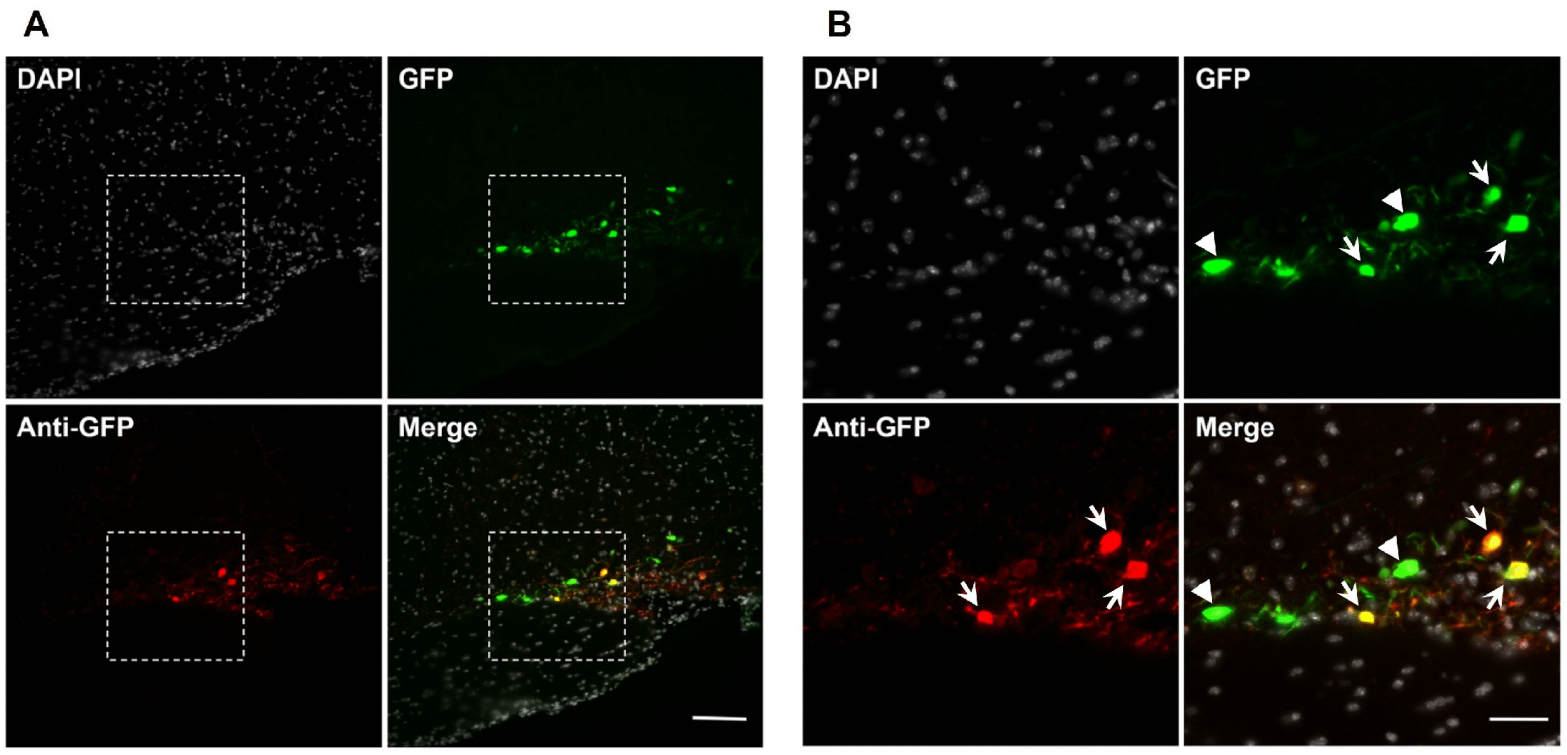
Colocalization of endogenous fluorescent GFP and immunostained GFP in neurons of the supraoptic nucleus (arrow) after intraperitoneal administration of 8.5% NaCl. (A) Neurons, containing: GFP only (green), GFP-immunopositive material (red), both GFP and GFP-immunopositive material (yellow at merge) with cell nuclei stained using 4’,6-diamidino-2-phenylindole (DAPI, gray). (B) The area outlined by the white dotted line in photo “A” at higher magnification: neurons, containing: GFP only (green, arrow head), GFP-immunopositive material (red), both GFP and GFP-immunopositive material (yellow at merge, arrow) with cell nuclei stained using DAPI (gray). Scale bars: main image – 100 µm; magnified area – 50 µm.

Colocalization of GFP and GFP-immunopositive material was assessed in SON neurons in mice, salt-loaded by intraperitoneal administration of 8.5% NaCl. All neurons contained GFP were immunopositive for GFP (Fig 6A, B).

### The number of tyrosine hydroxylase-immunopositive and green fluorescent protein neurons in the supraoptic nucleus of transgenic mice after salt loading

**Fig 7:**
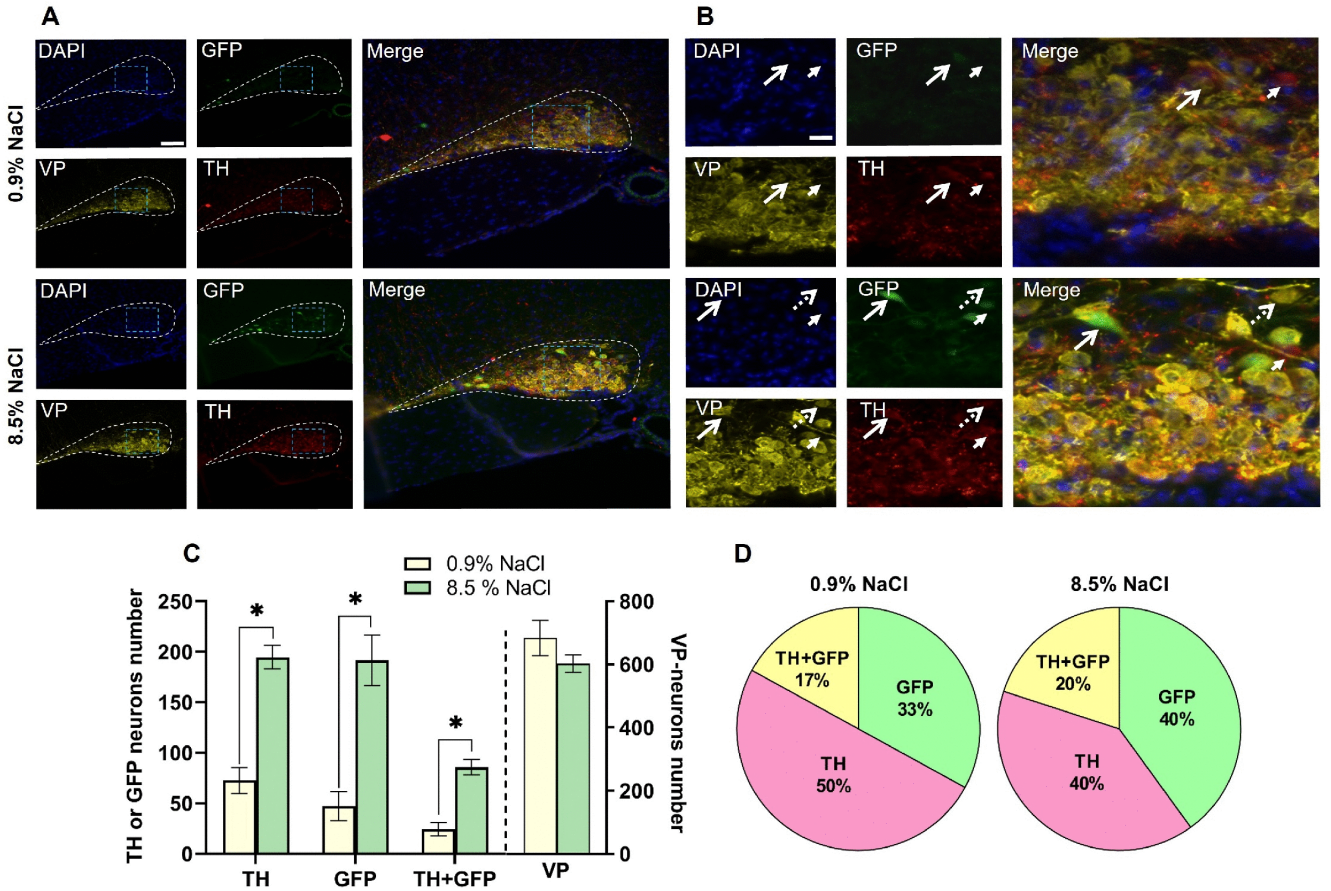
Vasopressin(VP)- and tyrosine hydroxylase(TH)-immunopositive neurons and green fluorescent protein(GFP) neurons in the supraoptic nucleus (SON) of transgenic mice [B6.B6D2-Tg(Th-EGFP)21−31Koba)] 10 hours after intraperitoneal administration of 8.5% NaCl in the experiment and 0.9% NaCl in the control. (A, B) TH-immunopositive neurons (red), VP-immunopositive neurons (yellow), and GFP neurons (green) with nuclei stained with 4′,6-diamidino-2-phenylindole (DAPI, blue) in the SON (dashed line) in the experiment and in the control at a low (A) and high (B) magnifications. Large open arrow – TH-immunopositive/GFP neurons, small closed arrow – TH-immunopositive/without GFP neurons, large dotted arrow – TH-immunonegative/GFP neurons. Scale bar: A – 100 µm, B – 20 µm. (C) Number of VP- and TH-immunopositive and/or GFP neurons in the SON in the experiment and in the control. (D) Diagrams of the proportions (in %) of neurons containing only TH-immunopositive material, only GFP, and simultaneously TH-immunopositive material and GFP in the SON of transgenic mice after intraperitoneal administration of 8.5% NaCl in the experiment (right) and 0.9% NaCl in the control (left) (n = 4 per group). The groups were compared for normality by using the Shapiro-Wilk test. Statistical analysis was performed using the one-way ANOVA multiple comparison test and post-hoc Tukey’s test, where * corresponds to statistically significant differences at p ≤ 0.05. The data are presented as mean ± SEM.

Ten hours after intraperitoneal injection of 8.5% NaCl (experiment) or 0.9% NaCl (control) to transgenic mice, the number of TH-immunopositive neurons and GFP neurons significantly increased in the SON of experimental animals compared to controls by 2.44 and 3.7 times, respectively (Fig 7A–C). A 3.5-fold increase in the number of TH-immunopositive neurons containing GFP was also observed (Fig 7C). In control mice, the populations of neurons stained only for GFP was 33%, stained only for TH – 50%, and double-stained for TH and GFP −17% (Fig 7D). In experimental mice, the population of neurons stained only for TH consisted of 40%, the population of neurons stained only for GFP was 40%, and the population of double-stained neurons was 20% (Fig 7D).

In the control group of transgenic mice, 10 hours after the injection of saline, the number of VP neurons in the SON (684 ± 55) did not differ significantly from that after intraperitoneal injection of 8.5% NaCl (602 ± 28) (Fig 7C).

### Expression of transcription factor genes in the supraoptic nucleus of C57BL/6 mice after salt loading

**Fig 8:**
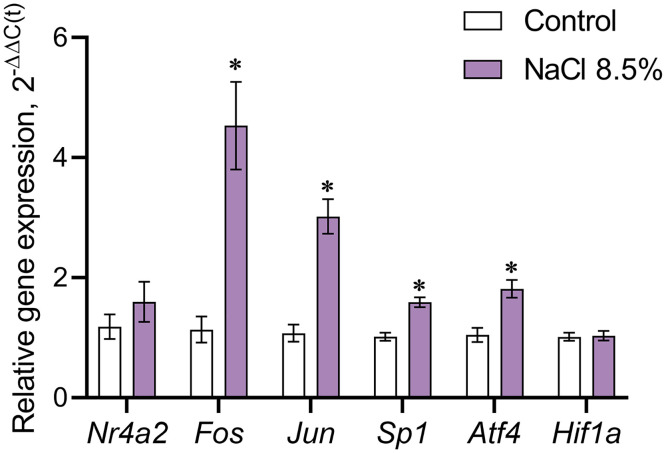
Expression of transcription factor genes in the supraoptic nucleus of mice 10 hours after intraperitoneal administration of 8.5% NaCl in the experiment and 0.9% NaCl in the control. The relative level of gene expression was estimated by the 2^-ΔΔC(t)^ method. The level of gene expression in the control was taken as 1. *Nr4a2* – gene of transcription factor Nurr1; *Fos* – gene of c-Fos; *Jun* – gene of c-Jun; *Sp1* – gene of transcription factor Sp1; *Atf4* – gene of activating transcription factor 4; *Hif1a* – gene of hypoxia-inducible factor 1-alpha. The groups (n = 12 per group) were compared for normality by using the Shapiro-Wilk test. Statistical analysis was performed using the paired t-test, where * corresponds to statistically significant differences at p ≤ 0.05, compared with the control. The data are presented as mean ± SEM.

Ten hours after intraperitoneal injection of 8.5% NaCl to mice, *Fos* expression in SON neurons increased by 4.53 times (p = 0.0006) and *Jun* by 3.02 times (p < 0.0001) compared to the control group (0.9% NaCl). In turn, the expression of *Sp1* increased by 1.6 times (p < 0.0001) and *Atf4* by 1.81 times (p = 0.0011), while the expression of *Nr4a2* and *Hif1a* did not change (Fig 8).

After 3 days of drinking 3% NaCl, no differences were found in the gene expression of all the transcription factors studied in the SON in mice compared to the control animals – drinking tap water (data not shown).

## Discussion

Based on previous experience of salt loading in rats and occasionally in mice [9,21,26,27], we first reproduced in mice the model of chronic salt loading by drinking a hypertonic saline solution. The SON was used for subsequent analysis as it contains only VPergic and OTergic neurons that synthesize TH upon salt loading. [17,18,28]. Although VPergic and OTergic neurons expressing TH are also contained in the paraventricular nucleus, we did not use this nucleus for analysis because it also contains other TH-expressing neurons, for example, those producing corticoliberin [27]. The most important result obtained when using 3% NaCl as a drink for 3, 4 and 5 days is that only after 3 days the number of TН-immunopositive neurons in the SON increased significantly, almost 30 times, compared to the control. However, according to semi-quantitative immunohistochemical analysis, the TH content in individual neurons in the experimental group did not change compared to the control.

In addition to the chronic salt loading model, we also reproduced the acute salt loading model, which, like the chronic model, has previously been frequently used mainly in rats and rather rarely in mice [14,18–21]. In this case, 8.5% NaCl was administered intraperitoneally, and TH expression was assessed 6, 10 and 24 hours later. In the control, 0.9% NaCl was administered according to the same scheme. The minimum period of 6 hours was chosen based on the fact that intracellular accumulation of a non-releasable protein, such as TH, could be detected no earlier than 5 hours after stimulation of its synthesis [29]. However, in our experiment, even 6 hours after the intraperitoneal injection of 8.5% NaCl, no reliable change in the number of TH-immunopositive neurons was observed compared to the control. It is important to emphasize that in this model, the number of TH-immunopositive neurons in mice, even in the control, was many times higher than in intact animals. This may be due to a stress reaction to the intraperitoneal injection procedure.

Ten hours after the intraperitoneal injection of 8.5% NaCl, the number of TH-immunopositive neurons in the SON increased 5-fold, compared to the control, exceeding 1.6-fold the number of TH-immunopositive neurons in mice that drank 3% NaCl for 3 days. Twenty-four hours after the intraperitoneal injection of 8.5% NaCl, the number of TH-immunopositive neurons in the SON decreased to the control level. This may be related to the enzymatic degradation of TH. This hypothesis could be tested by assessing the kinetics of TH degradation and thus determining the half-life of this protein. Unfortunately, such data have not yet been obtained, and this is the subject of further research.

Based on a comparison between the number of TH-immunopositive SON neurons after drinking 3% NaCl for 3 days and the same parameter 10 hours after an intraperitoneal injection of 8.5% NaCl, it becomes obvious that the second model meets much better our requirements formulated above. An additional advantage of the second model over the first one is the increased intraneuronal TH content.

We managed not only to develop an optimal salt loading model according to TH expression in SON neurons in wild-type C57BL/6 mice by intraperitoneal injection of 8.5% NaCl, but also to test this intervention on transgenic B6.B6D2-Tg(Th-EGFP)21–31Kobа mice, in which the expression of the GFP gene is under the control of the TH promoter [21]. This allowed us to simultaneously assess in transgenic mice TH gene (*Th*) expression by the presence of GFP and TH synthesis by immunostaining. Three types of neurons were distinguished based on these indicators. Some neurons contained only GFP, which can probably be explained by TH level beyond the resolution of the immunohistochemistry we used. As shown previously, TH-immunopositive material is also not visible in some neurons of the substantia nigra and striatum that most probably express this protein [21,30,31]. This is apparently due to the low resolution of the immunohistochemistry we used.

It also cannot be excluded that TH synthesized in the cell bodies of SON neurons is immediately transported to axons, which is typical for catecholaminergic neurons [32]. If this hypothesis is valid, the use of transgenic mice expressing GFP under the TH gene promoter could expand the possibilities for assessing the mechanisms of TH synthesis in SON neuron cell bodies, for example, by promoting TH accumulation in neuronal cell bodies after inhibition of axonal transport. It also cannot be ruled out that differences in the colocalization of TH and GFP in the cell bodies of the SON neurons may be due to ectopic expression of the transgene, which was previously shown for the substantia nigra neurons [22]. Other neurons contained both substances, TH-immunopositive material and GFP, which indicates not only the expression of *Th*, but also the synthesis and accumulation of TH. The discovery of a third population of neurons containing TH-immunopositive protein in the absence of GFP was unexpected. Indeed, it is difficult to understand why TH synthesis occurs in the absence of GFP, as an indicator of *Th* expression. Hypothetically, this may be due to a weak fluorescence of GFP, undetectable by a fluorescence microscope. Moreover, according to previous studies, tissue fixation can reduce GFP fluorescence [33]. To exclude the effect of 4% PAF fixation on GFP fluorescence, we used immunohistochemistry to colocalize GFP and GFP-immunoreactive protein. Despite our concerns, we observed complete overlap of GFP and GFP-immunoreactive protein in a number of neurons, confirming the absence of a significant effect of fixation on GFP fluorescence. Although the absence of GFP in some TH-immunopositive neurons may be due to rapid GFP degradation, but this is unlikely. Indeed, as Corish and Tyler-Smith showed, the half-life of GFP averages 26 hours [34], which certainly is longer than the period of our experiment for 10 hours. However, it cannot be excluded that that under the rat *Th* promoter in B6.B6D2-Tg(Th-EGFP)21–31Kobа mice, not all the transcription factors necessary for triggering *Th* expression are expressed. This issue can be later solved by a comparative analysis of gene expression in individual fractions of the above three types of neurons.

When using the salt loading model with an intraperitoneal administration to B6.B6D2-Tg(Th-EGFP)21–31Kobа mice of 8.5% NaCl in the experiment and 0.9% NaCl in the control, a multiple (x 2.4–3.7) increase in the number of SON neurons containing TH-immunoreactive material and GFP was shown. This indicates that the increase in TH production in osmotically stimulated neurons is accompanied by a significant increase in *Th* expression. Previously, we discovered similar changes in *Th* expression and TH synthesis in DAergic neurons of the substantia nigra due to a compensatory increase in their functional activity [35].

In addition to demonstrating an increase in TH synthesis following intraperitoneal administration of hypertonic NaCl, it was important to determine the intracellular regulation of *Th* expression. In this context, an analysis of the expression of transcription factor genes showed that 10 hours after the administration of 8.5% NaCl, the expression of the *c-Fos* and *c-Jun*, which encode the transcription factor AP-1, was increased. These data are consistent with the fact that the *Th* promoter contains a binding site for AP-1, and the position of the binding site in the promoter is strictly determined in rats, mice, and humans [36–38]. Our results are also consistent with a previous study showing that Fos expression is increased in the SON of rats under salt loading [13,18–20]. Based on the above, we can conclude that the increase in *Th* expression in SON neurons observed under salt loading is due to the synthesis of c-Fos and c-Jun, the formation of the AP-1 complex, and its binding to the *Th* promoter. In addition to the increase in *Fos* and *Jun* expression, we have demonstrated under salt loading an increase in the expression of the transcription factor Sp1 gene (*Sp1*), the landing site of which – the GC box or Sp1 motif – was previously found on the *Th* promoter [39,40].

Unfortunately, studies on the regulation of *Th* expression via the Sp1 motif are extremely limited. It is only known that regulation of *Th* expression via Sp1 binding to the Sp1 motif of the *Th* promoter occurs in animals not exposed to stress, whereas during immobilization stress, Egr1 binds to the Sp1 motif. Importantly, the binding site of Egr1 significantly overlaps with the binding site of Sp1 [41]. In addition, it is known that Egr1 binding to the Sp1 motif can modulate the regulation of *Th* transcription by AP1 factors [42]. The increase in *Fos*, *Jun*, and *Sp1* expression that we observed may indicate that *Th* transcription in the SON is activated by AP-1 and can be regulated by Sp1 binding to the Sp1 motif in a manner similar to that observed with Egr1 binding to the Sp1 motif. However, this hypothesis requires further experimental verification.

Under salt loading by intraperitoneal administration of 8.5% NaCl, we also found increased expression of *Atf4* in SON neurons, which is considered an indicator of endoplasmic reticulum stress [43]. Indeed, endoplasmic reticulum stress is accompanied by a disruption of protein homeostasis, which leads to the accumulation of misfolded and unfolded proteins in the endoplasmic reticulum [44]. Based on the above, an increase in *Atf4* expression in the SON in response to salt loading indicates the development of endoplasmic reticulum stress.

The *Th* promoter contains a binding site for hypoxia-inducible factor 1-alpha [45]. We failed to detect any changes in the expression of *Hif1a* or *Nr4a2* under salt loading. It should be noted that *Nr4a2* encodes the Nurr1 protein, a *Th* transcription factor [40] that plays an important role in the development, regulation, and survival of DAergic neurons and maintaining their phenotype [46]. No change in the expression of *Hif1a* and *Nr4a2* indicates that the transcription factors they encode, unlike AP-1 and activating transcription factor 4, do not affect *Th* expression in the SON under salt loading.

Thus, the increase in *Th* expression in SON neurons, associated with an increase in *Fos*, *Jun*, *Atf4*, and *Sp1* expression, is most likely a manifestation of non-specific cell activation and the result of endoplasmic reticulum stress, induced by salt loading.

## Conclusions and prospects

The following results were obtained in this work:

1. Two models of salt loading, previously developed in rats, were reproduced in mice: (i) a chronic model using 3% NaCl as a drink for several days, (ii) an acute model with intraperitoneal administration of 8.5% NaCl and obtaining material during the same day.
2. It was shown that after 3 days of drinking 3% NaCl, the number of TH-containing neurons in the SON increased significantly compared to the control (tap water), but the intraneuronal TH content did not change.
3. It was shown that 10 hours after intraperitoneal administration of 8.5% NaCl, the number of neurons producing TH, in the SON significantly increased compared to the control (administration of 0.9% NaCl), which was accompanied by an increase in TH content in individual neurons.
4. Comparative analysis of the first and second models of salt loading by assessing the level of TH synthesis showed greater efficiency of the second model, which was chosen for further research.
5. It has been shown that intraperitoneal administration of a hypertonic solution to mice is accompanied by an increase in the expression of the *Th* and transcription factors involved in the regulation of this gene, in the neurons of the SON.

In further studies, it is desirable to determine in a mouse model of salt loading caused by intraperitoneal administration of hypertonic saline:

1. Does TH in SON neurons have enzymatic activity and synthesize L-DOPA;
2. Whether SON neurons, in addition to TH, express other features of the DAergic phenotype.

## Supporting information

S1 File IHC protocol. Laboratory protocol for processing material for immunohistochemistry.
(PDF)

S2 File PCR protocol. Laboratory protocol for RNA extraction and polymerase chain reaction quantification.
(PDF)

S3 File Raw Data. (XLSX)
