# Supplementary material for: Salt loading as a promising approach to study the dopaminergic phenotype of neurons of the supraoptic nucleus in mice
Source: PLoS One. 2026 Feb 6;21(2):e0340281. doi: 10.1371/journal.pone.0340281 (PMC12880680; doi:10.1371/journal.pone.0340281)
Supplement: S1 File — Laboratory protocol for processing material for immunohistochemistry. (PDF) [file pone.0340281.s001.pdf]

Laboratory protocol for processing material for immunohistochemistry

Basic laboratory protocol for double immunostaining of neurons for tyrosine hydroxylase and vasopressin.

Reagents:

1. Alexa-Fluor-488 donkey antibodies against sheep gamma globulins (Invitrogen, USA, A11015)
2. Alexa-Fluor-555 donkey antibodies against rabbit gamma globulins (Invitrogen, USA, A32794)
3. Bovine serum albumin (Sigma-Aldrich, USA)
4. HCl 37% (Sigma-Aldrich, USA)
5. Hexane (Sigma-Aldrich, USA)
6. Isoflurane (Laboratorios Karizoo, Spain)
7. Liquid N_2
8. Medium containing 4′,6-diamidino-2-phenylindole (DAPI) (Abcam, USA)
9. Na_2HPO_4 (Sigma-Aldrich, USA)
10. NaCl (Sigma-Aldrich, USA)
11. NaH_2PO_4 (Sigma-Aldrich, USA)
12. NaOH 50% (Sigma-Aldrich, USA)
13. Paraformaldehyde (Sigma-Aldrich, USA)
14. Rabbit monoclonal antibodies to vasopressin (VP) (Abcam, USA, AB 213708)
15. Sheep polyclonal antibodies to tyrosine hydroxylase (TH) (Millipore, USA, AB 1542)
16. Sucrose (Sigma-Aldrich, USA)
17. Tissue freezing medium (Leica Biosystems Richmond, Inc., the United Kingdom)
18. Triton X-100 solution (Sigma-Aldrich, USA)

Preparation of solutions:

1. **1M NaOH.**

   Add 8 ml of 50% NaOH to 92 ml of distilled water. The resulting solution can be stored in a tightly closed glass bottle at room temperature for 1 month.

2. **1M HCl**.

   Add 9.85 ml of 37% HCl to 90.15 ml of distilled water. The resulting solution can be stored in a tightly closed glass bottle at room temperature for 1 month.90.15 мл дистиллированной воды добавить 9.85 мл 37% HCl..

3. **0.2 M phosphate buffer (PB)** mM: Na_2HPO_4 161.55; NaH_2PO_4 38.33 (pH = 7.2-7.4).

   Dissolve 22.94 g of Na2HPO4 and 4.6 g of NaH2PO4 in 1 liter of distilled water. Check the pH of the resulting solution and, if necessary, adjust to 7.2-7.4 with 1 N NaOH or 1 N HCl. The resulting solution can be stored in a tightly closed bottle at 4°C for 1 month. Before use, warm the solution to room temperature and check the pH. If the pH differs from 7.2-7.4, do not use the solution.

4. **4% Paraformaldehyde in 0.1 M phosphate buffer (4% PAF)**.

   Add 80 g of PAF to 1 liter of distilled water (8% PAF). Heat this solution to 70°C, stirring with a magnetic stirrer. When large flocs form, add a few drops of 1N NaOH until the solution becomes clear. Cool the resulting solution to room temperature. The prepared 8% PAF can be stored in a tightly closed flask at 4°C for 2 weeks. Before use, dilute 8% PAF with 0.2 M FB in a 1:1 ratio and cool the resulting solution (4% PAF to 0.1 M FB) to 4°C.

5. **0.02 M phosphate-buffered saline (PBS)** (pH = 7.2-7.4) mM: Na_2HPO_4 16.155; NaH_2PO_4 3.833; NaCl 154 (pH = 7.2-7.4).

   Add 100 ml of 0.2 M sodium phosphate buffer to 900 ml of distilled water and dissolve 9 g of NaCl. Check the pH of the resulting solution and, if necessary, adjust it to 7.2–7.4 with 1 N NaOH or 1 N HCl. The resulting solution can be stored in a tightly closed bottle at 4°C for 1 month. Before use, warm the solution to room temperature and check the pH. If the pH differs from 7.2–7.4, do not use it.

6. **20% sucrose** in 0.02 M PBS.

   Before use, dissolve 20 g sucrose in 100 ml 0.02 M PBS.

7. **10% Triton X-100**.

   In a 15 ml plastic tube, add 1 ml of Triton X-100 to 9 ml of distilled water and dissolve by shaking on a shaker. The resulting solution can be stored in a tightly closed glass bottle at room temperature for 12 months.

8. **8. Solution for inhibition of non-specific binding of antibodies: 3% Bovine serum albumin (BSA) and 0,3% Triton X-100 in 0.02 M PBS (pH = 7.2-7.4).**

   Before use, warm 0.02 M PBS to room temperature and check the pH (7.2–7.4). In a 5 ml plastic tube, dissolve 120 mg BSA in 4 ml of 0.02 M PBS at vortexing. Then add 120 μl of 10% Triton X-100 solution.

9. **Basic solution for incubation with primary antibodies: 1% bovine serum albumin (BSA) and 0.1% Triton X-100 in 0.02 M PBS (pH = 7.2-7.4).**

   Before use, warm 0.02 M PBS to room temperature and check the pH (7.2–7.4). In a 2 ml Eppendorf tube, prepare “solution for inhibiting nonspecific antibody binding» in 0.02 M PBS at a ratio of 1:2.

10. **Preparation of a solution of primary antibodies to vasopressin and tyrosine hydroxylase**

    20 minutes before use, add 1.2 μl of rabbit monoclonal antibodies to VP (1:500) and 0.857 μl of sheep polyclonal antibodies to TH (1:700) to 1.5 ml of Eppendorf in 600 μl of “incubation solution with 1 antibody”.

11. **Preparation of a solution of secondary antibodies for detection of antibodies to vasopressin on sections.**

    Twenty minutes before use, add 0.6 μl Alexa-Fluor-555 goat anti-rabbit gamma globulin antibody (1:1000) to 1.5 ml Eppendorf in 600 μl 0.02 M PBS (pH = 7.2-7.4).

12. **Preparation of a solution of secondary antibodies for detection of antibodies to tyrosine hydroxylase on sections.**

    Twenty minutes before use, add 0.6 μl of Alexa-Fluor-488 donkey anti-sheep gamma globulin antibody (1:1000) to 1.5 ml of Eppendorf in 600 μl of 0.02 M PBS (pH = 7.2-7.4).

Stages of work

1. **Brain dissection in mice.**

Mice were anesthetized with 2.4% isoflurane (Laboratorios Karizoo, Spain) using a SomnoSuite anesthesia device (Kent Scientific, USA). The mice were then decapitated, and their brains were removed.

2. **Fixation and freezing of the brain.**

The brains were fixed in 4% paraformaldehyde prepared in 0.2 M phosphate buffer (pH 7.2–7.4) for 12 hours at 4°C. After rinsing in 0.02 M phosphate-buffered saline (PBS) (pH 7.2–7.4) three times for 30 minutes each, the brains were incubated in 20% sucrose in 0.02 M PBS for 24 hours at 4°C. The brains were then transferred to a plastic container (150 ml) containing hexane, preliminary cooled with liquid nitrogen to -40°C, and kept there for 1 minute. The frozen brains were transferred with cooled tweezers into preliminary cooled 5 ml plastic tubes, which were stored at -70°C until immunohistochemistry.

3. **Preparation of frozen brain sections on a Leica CM1950 cryostat**

The cryostat chamber was cooled to -20°C, and the brain holder was cooled to -19°C. The brain was glued to the holder using tissue freezing medium. Serial brain sections, 12 μm thick, were prepared at the level of the supraoptic nucleus. For subsequent immunostaining, every third section was mounted on slides at a distance from -0.46 mm to -1.06 mm in the rostrocaudal direction according to the mouse brain atlas [22].

4. **Immunocytochemistry**

a. Inhibition of non-specific binding of antibodies

Put 300 μl of "non-specific antibody binding inhibiting solution" to each slide and incubate for 1 hour at 20°C. Short rinsing

b. Incubation with primary antibodies to vasopressin and tyrosine hydroxylase.

Put 200 μl of a solution (1% BSA with 0.1% Triton X-100 in 0.02 M PBS) with primary antibodies (rabbit monoclonal antibodies to VP and sheep polyclonal antibodies to TH) to each slide and incubate for 20 hours at 20 °C. Short rinsing.

c. Thorough rinsing.

The sections are washed by immersing slides in a bath with PBS and shaking them on a shaker for 10 minutes at 20°C. This procedure is repeated three times.

d. Incubation with secondary antibodies at vasopressin immunostaining

Put 200 µl of a solution (0.02 M PBS) containing Alexa-Fluor-555 goat antibodies against rabbit gamma globulins to each slide. Incubate for 2 hours at 20°C. Short rinsing.

e. Thorough rinsing.

The sections are washed three times by immersing slides in a bath with PBS and shaking them on a shaker for 10 minutes at 20°C. This procedure is repeated three times.

f. Incubation with secondary antibodies at tyrosine hydroxylase immunostaining

Put 200 µl of a solution (0.02 M PBS) containing Alexa-Fluor-488 donkey antibodies against sheep gamma globulins to each slide and incubate sections for 2 hours at 20°C. Short rinsing. .

g. Thorough rinsing.

The sections are washed by immersing slides in a bath with PBS and shaking them on a shaker for 10 minutes at 20°C. This procedure is repeated three times.

h. Section mounting.

Put a few drops of DAPI-containing medium to each slide with sections, ensuring all sections are covered. Wait 1 minute and cover sections with a coverslip. The mounting medium is polymerized for 12 hours. Store sections in the dark.
