# Supplementary material for: Salt loading as a promising approach to study the dopaminergic phenotype of neurons of the supraoptic nucleus in mice
Source: PLoS One. 2026 Feb 6;21(2):e0340281. doi: 10.1371/journal.pone.0340281 (PMC12880680; doi:10.1371/journal.pone.0340281)
Supplement: S2 File — Laboratory protocol for RNA extraction and polymerase chain reaction quantification. (PDF) [file pone.0340281.s002.pdf]

Laboratory protocol for RNA extraction and quantitative polymerase chain reaction

The basic protocol used in the laboratory for RNA extraction and qPCR. The expression of transcription factor genes was determined in mice 10 hours after intraperitoneal administration of 8.5% NaCl in the experiment and 0.9% NaCl in the control, as well as 3 days after drinking 3% NaCl in the experiment and tap water in the control (n = 12 per group).

Reagents:

- TRI Reagent (Sigma-Aldrich);

  Caution: This substance is toxic, so avoid direct exposure or inhalation.
- 1-Bromo-3-chloropropane (Sigma-Aldrich);
- Isopropyl alcohol (Sigma-Aldrich);
- Glycogen, RNA grade (Thermo Fisher Scientific);
- Ethanol 95% (Acros Organics);
- PCR grade water (Evrogen);
- DNase I, RNase-free (Thermo Fisher Scientific);
- MMLV RT synthesis kit (Evrogen, Moscow, Russia);
- qPCRmix-HS SYBR+LowROX (Evrogen);
- Oligonucleotide primers (Evrogen; Table 1).

Table 1. Oligonucleotide primers used for quantitative polymerase chain reaction.

Gene	Protein	Forward primer	Reverse primer
Cyc1	Cytochrome C1	GCGGCCAGGGAAGTTGT	GCCAGTGAGCAGGGAAAATAC
Th	Tyrosine hydroxylase	TCAGAGGAGCCCGAGGTC	GGGCGCTGGATACGAGAG
Avp	Arginine vasopressin	CCCAAGAGGCGGCAAGAG	CAGGGCGAGGGCAGGTAG
Nr4a2	Transcription factor Nurr1	*CCGAAGAGCCCACAGGAT*	*CCATAGAGCCGGTCAGGAG*
Fos	c-Fos	*AGAGCGCCCCATCCTTAC*	*GCTCTACTTTGCCCCTTCTG*
Jun	c-Jun	*CGCCCCTGTCCCCTATC*	*TAAGCTGTGCCACCTGTTCC*
Sp1	Transcription factor Sp1	*GGCCTTGCTAATAATGTGCTCT*	*CATGTTGCTGGTGGTAGTAGTTGT*
Atf4	Activating transcription factor 4	*CTTATGACCCACCTGGAGTTAGT*	*CTAGTGGCTGCTGTCTTGTTTT*
Hif1a	Hypoxia-inducible factor 1-alpha	*ACATGATGGCTCCCTTTTTC*	*CTCCGTTCCATTCTGTTCACT*

Step-by-step experimental procedures:

1. **Extraction of the total RNA from SON samples**

a. 1000 μl TRI Reagent was added to the SON tissue. This mixture was incubated for 5 min at 20°C and homogenized by pipetting.

b. 100 μl of 1-bromo-3-chloropropane were added to the homogenized samples and incubation was continued for 15 min at 20°C with vortexing every 3 min.

c. Phases were separated by centrifugation at 21,000× g for 15 min at 4°C.

d. The aqueous phase containing RNA was transferred to a new 1.5 ml centrifuge tube and 500 μl isopropyl alcohol was added.

e. For better RNA precipitation, 1 μl glycogen was added to aqueous phase.

f. The sample was incubated with shaking for 10 min at 20°C, followed by RNA precipitation by centrifugation at 21,000×g for 10 min at 4°C.
g. The sample was incubated with shaking for 10 min at 20°C, followed by RNA precipitation by centrifugation at 21,000× g for 10 min at 4°C.
h. The supernatant was removed.
i. The precipitate was washed three times in 1 ml 80% ethanol and centrifuged at 21,000× g for 10 min at 4°C.
j. After the last centrifugation, ethanol was removed and the RNA precipitate was air-dried for 15 min.
k. RNA was dissolved in 20 µl PCR grade water.
l. The RNA concentration in all samples was measured using a NanoDrop 8000 (Thermo Fisher Scientific, USA).

2. **Removal of genomic DNA**

a. The remaining genomic DNA was removed using DNase I RNase-free (Thermo Fisher Scientific, USA) according to the manufacturer's recommendations.

3. **Reverse transcription reaction**

a. Complementary DNA was synthesized from 300 ng of RNA using the MMLV RT synthesis kit (Evrogen, Moscow, Russia) according to the manufacturer's recommendations.

4. **qPCR**

a. qPCR was performed on a QuantStudio 12k Flex thermocycler (Applied Biosystems, Waltham, USA) using the qPCRmix-HS SYBR + LowROX reaction mixture (Evrogen, Moscow, Russia). Oligonucleotide primers (Evrogen, Moscow, Russia) are exposed in Table 1.
b. For qPCR, 500 ng complementary DNA was used.
c. Amplification was carried out according to the following protocol:
    - hold stage – at 50°C for 2 min and then at 95°C for 10 min;
    - PCR stage – at 95°C for 15 s and then at 60°C for 1 min (40 cycles);
    - melt curve stage – sequentially at 95°C for 15 s, at 60°C for 1 min, and at 95°C for 15 s.

5. **Gene expression analysis**

a. *Cyc1* was used as a housekeeping gene.
b. Gene expression was assessed using the $2^{-\Delta\Delta Ct}$ method:

Formulas (1) and (2) were used for calculating ΔΔCt as follows:

$$\Delta Ct = (Ct(\textit{gene}) - Ct(\textit{Cyc1}))$$

$$\Delta\Delta Ct = (\Delta Ct(\text{sample}) - \Delta Ct(\text{medium control}))$$

The results were calculated as the geometric mean of the group and are presented as fold changes with respect to the control.

c. *Avp* expression level was used to determine the accuracy of SON dissection from the brain sections.
d. Samples with low *Avp* expression levels were excluded from the analysis.
